# Effect of almond consumption on vascular function in patients with coronary artery disease: a randomized, controlled, cross-over trial

**DOI:** 10.1186/s12937-015-0049-5

**Published:** 2015-06-17

**Authors:** C-Y. Oliver Chen, Monika Holbrook, Mai-Ann Duess, Mustali M Dohadwala, Naomi M Hamburg, Bela F. Asztalos, Paul E. Milbury, Jeffrey B. Blumberg, Joseph A. Vita

**Affiliations:** 1 Jean Mayer USDA Human Nutrition Research Center on Aging, Tufts University, Boston, MA USA; 2 Evans Department of Medicine and the Whitaker Cardiovascular Institute, Boston University School of Medicine, Boston, MA USA; 3 Antioxidants Research Laboratory, Jean Mayer USDA Human Nutrition Research Center on Aging at Tufts University, 711 Washington Street, Boston, MA 02111 USA

**Keywords:** Almonds, Coronary arterial disease, Dietary quality, Endothelial function, Inflammation, Oxidative stress

## Abstract

**Objective:**

Almonds reduce cardiovascular disease risk via cholesterol reduction, anti-inflammation, glucoregulation, and antioxidation. The objective of this randomized, controlled, cross-over trial was to determine whether the addition of 85 g almonds daily to a National Cholesterol Education Program (NCEP) Step 1 diet (ALM) for 6 weeks would improve vascular function and inflammation in patients with coronary artery disease (CAD).

**Research design and methods:**

A randomized, controlled, crossover trial was conducted in Boston, MA to test whether as compared to a control NCEP Step 1 diet absent nuts (CON), incorporation of almonds (85 g/day) into the CON diet (ALM) would improve vascular function and inflammation. The study duration was 22 weeks including a 6-weeks run-in period, two 6-weeks intervention phases, and a 4-weeks washout period between the intervention phases. A total of 45 CAD patients (27 F/18 M, 45–77 y, BMI = 20-41 kg/m^2^) completed the study. Drug therapies used by patients were stable throughout the duration of the trial.

**Results:**

The addition of almonds to the CON diet increased plasma α-tocopherol status by a mean of 5.8 %, reflecting patient compliance (P ≤0.05). However, the ALM diet did not alter vascular function assessed by measures of flow-mediated dilation, peripheral arterial tonometry, and pulse wave velocity. Further, the ALM diet did not significantly modify the serum lipid profile, blood pressure, C-reactive protein, tumor necrosis factor-α or E-selectin. The ALM diet tended to decrease vascular cell adhesion molecule-1 by 5.3 % (*P* = 0.064) and increase urinary nitric oxide by 17.5 % (*P* = 0.112). The ALM intervention improved the overall quality of the diet by increasing calcium, magnesium, choline, and fiber intakes above the Estimated Average Requirement (EAR) or Recommended Dietary Allowance (RDA).

**Conclusions:**

Thus, the addition of almonds to a NECP Step 1 diet did not significantly impact vascular function, lipid profile or systematic inflammation in CAD patients receiving good medical care and polypharmacy therapies but did improve diet quality without any untoward effect.

**Trial registration:**

The trial was registered with the ClinicalTrials.Gov with the identifier: NCT00782015.

## Background

Coronary artery disease (CAD) is one of the most common causes of death in middle- and high-income countries [1]. Patients with CAD typically receive several medications as part of their treatment to protect against recurrent cardiac events and all-cause mortality. Lifestyle modifications comprised of regular participation in physical activity, diets low in fat and rich in plant foods and smoking cessation are recommended to complement polypharmacy regimens in the management of CAD patients [2].

**Fig. 1:**
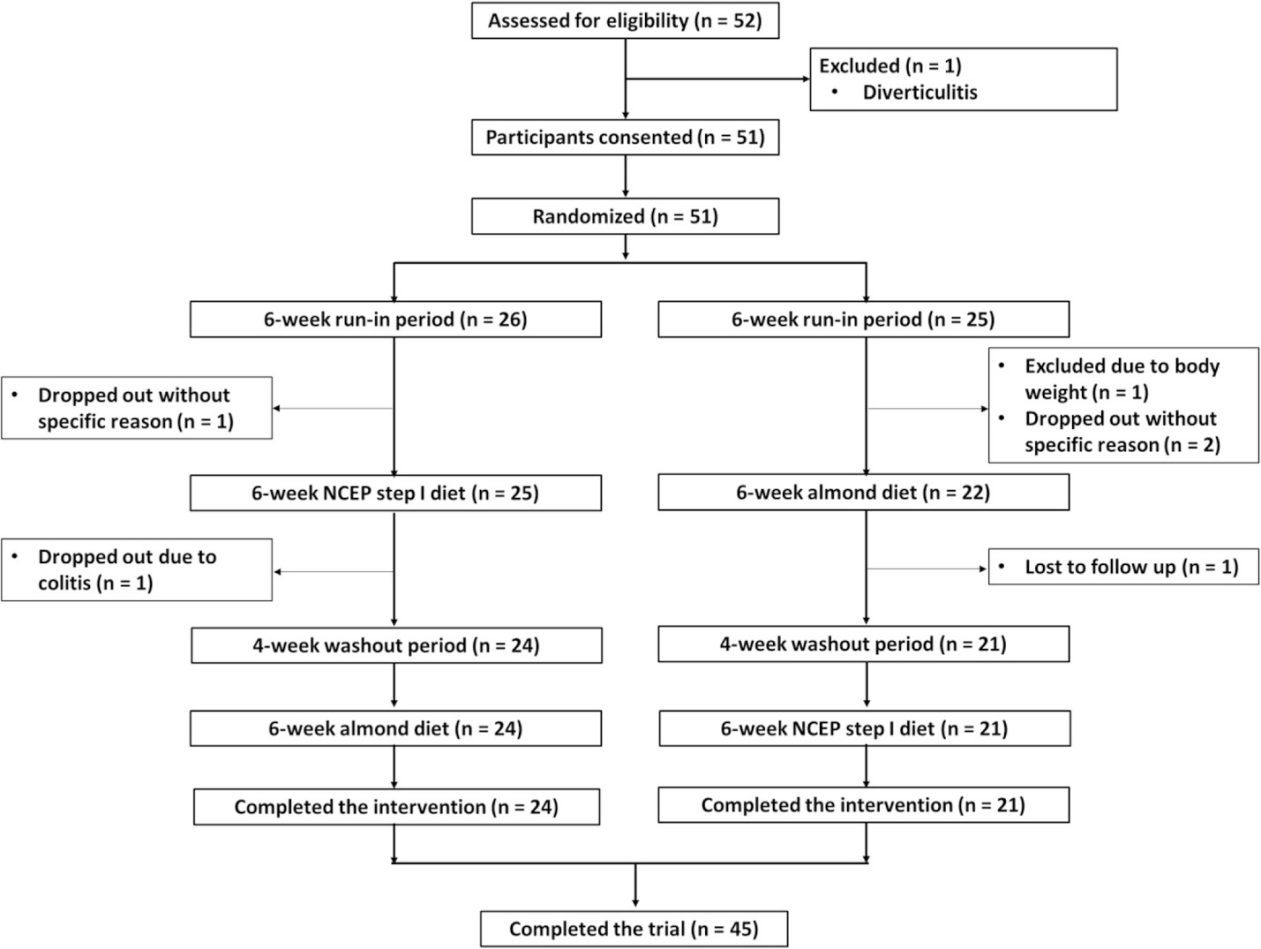
CONSORT flow diagram

Patients with CAD commonly have higher circulating cholesterol levels than their clinically healthy counterparts [3, 4]. Thus, patients with CAD are almost always treated with a hypocholesterolemic drug, usually a statin, and provided with recommendations for lifestyle modification for secondary prevention. Different dietary patterns, including the Mediterranean, DASH, and Portfolio Eating Plan diets, can complement statin therapy as well as provide other cardioprotective benefits [5, 6]. The American Heart Association recommends a diet rich in nuts, fruits and vegetables, and low in saturated fats for both primary and secondary prevention of CAD [7]. The Lyon Diet Heart Study was the first clinical trial to demonstrate that the Mediterranean diet is protective in the secondary prevention of CAD [8].

Among the variety of plant foods that may promote heart health, tree nuts are high in fiber, unsaturated fat, selected vitamins and minerals, and phytochemicals. This nutrient profile contributes to the observed reduction in risk of cardiovascular disease (CVD) among those who frequently consume nuts [9, 10]. Recently, the PREDIMED trial found that a Mediterranean diet supplemented with 30 g/day tree nuts (almonds, hazelnuts, and walnuts) decreased risk of cardiovascular events by 28 % [11]. Among tree nuts, almonds are rich in monounsaturated fat, fiber, α-tocopherol, copper and magnesium, and phytochemicals including phytosterols and polyphenols. Research on almonds suggests they may have beneficial actions on serum cholesterol, body weight, glucose homeostasis, inflammation, and oxidative stress [12, 13].

In addition to their macronutrient profile, almonds provide a good source of nutrients known to modulate vascular function, including L-arginine, flavonoids, folic acid, and vitamin E. In a clinical trial of 60 healthy men with risk factors for CVD, Choudhury et al. [14] tested 50 g almonds/day for 4 weeks and found improved flow-mediated dilatation (FMD). However, no studies are available that examine the effect of almonds on endothelial reactivity in patients with compromised vascular function. Therefore, we hypothesized that addition of 85 g/day almonds to a National Cholesterol Education Program (NCEP) Step 1 diet would improve vascular function in patients with CAD via an increase in nitric oxide (NO) availability and reduction in dyslipidemia, systemic inflammation, and/or oxidative stress.

## Methods and materials

### Subjects

Patients with angiographically proven CAD were recruited from the cardiology practice at the Boston Medical Center in Boston, MA Fig. 1 Consecutive patients with CAD coming to the cardiology practice were assessed for eligibility. CAD was confirmed with obstructive lesion(s) on angiogram, history of myocardial infarction, and/or positive stress test. The medical record was also reviewed through the Boston Medical Center electronic medical health record. Other inclusion criteria used to screen eligibility also included: age, 21–80 y; body mass index (BMI), 20–41 kg/m^2^; body weight, <115 kg; willingness and ability to provide written informed consent; and the ability to understand, participate, and comply with study requirements. The exclusion criteria included: women who are pregnant or planning to become pregnant; clinical history of other major illness including end-stage cancer, renal failure, hepatic failure or other conditions that in the opinion of the study physician make a clinical study inappropriate; treatment with an investigational new drug within the last 30 day; history of a psychological illness or condition; taking dietary supplements (including multivitamins and herbal supplements); and eating any nuts within 1 month of enrollment. Subjects were asked to withhold all vasoactive medications (nitrates, calcium channel blockers, beta blockers, angiotensin converting enzyme inhibitors, and other vasodilators) for 24 h prior to each ultrasound measurement. In our previous study [15], we reported that patients with stable CAD were able to withhold vasoactive medications for 24 h prior to the ultrasound tests without observed adverse effects. Further, a physician was available to determine eligibility and in case of emergencies. The study design was approved by the Institutional Review Boards of Tufts University Health Sciences Campus, Tufts Medical Center, and Boston Medical Center. All participants signed a written informed consent agreement before participating. The study was registered in the ClinicalTrials.gov, and the registration number is “NCT00782015”.

### Study design

A randomized, controlled, crossover trial was conducted to test the hypothesis that as compared to a control NCEP Step 1 diet absent nuts (CON), the incorporation of almonds (85 g/day) into the CON diet (ALM) would improve endothelial function, diminish inflammation, and/or oxidative stress. The dose of almonds was selected because Jenkins et al. [16] illustrated a dose- dependent effect on risk factors of cardiovascular disease and the amount of almonds would have a marked impact on nutrition quality of subjects. After eligible subjects were enrolled, they were randomized to one of the study sequences (ALM-CON or CON-ALM) using randomly permuted blocks of size 6 (total 10 blocks). The program in the randomization.com was employed for the randomization. After a 6 weeks run-in period, the patients began to consume one of the diets for 6 weeks and then the alternate diet for the other 6 weeks period with a 4-weeks washout period before the switch. During the run-in and washout periods, all subjects were asked to consume the CON diet. Prior to the start of the trial, all participants were counseled about the CON diet by a registered dietitian and on issues concerning inclusion of almonds to the CON diet. They were instructed to consume almonds as snack in the morning and afternoon. They were also instructed to replace almonds with other food items to maintain constant energy intake during the trial. Nutrient intakes during the trial were assessed with 3 food recall questionnaires conducted at the study entry (before the run-in) and the end of each intervention phase. Participants were contacted via frequent phone calls to encourage compliance to the study diets. Further, package bags were collected to confirm compliance at the end of the almond intervention phase. Raw whole almonds were generously pre-packaged at ~28.4 g servings and provided by the Almond Board of California.

### Vascular reactivity

During the study, subjects visited the study site (Vascular Research Unit, Boston Medical Center) for 4 times after an overnight fast for assessment of vascular reactivity. After their arrival in the morning, vital signs, including blood pressure and body weight, were measured, followed by urine and blood collections. Vascular reactivity was assessed based on flow-mediated dilation (FMD), peripheral arterial tonometry (PAT), and pulse wave velocity (PWV). After blood and urine collections, subjects rested quietly in a recumbent position for 10 min. Subsequently, arterial blood pressure was determined using an automated physiologic recorder (Dinamap, GE Health Care, Piscataway, NJ), followed by assessment of FMD in brachial artery and fingertip vessels as described previously [17]. Brachial artery ultrasound was used to determine FMD and hyperemic flow measured in the brachial artery. Briefly, a 2-dimensional and Doppler ultrasound image was recorded before and 1 min after induction of reactive hyperemia by 5-min cuff occlusion in the upper arm. Simultaneously, endothelial function in the fingertip vessels was measured by using a digital pulse amplitude tonometry (Endo-PAT, Itamar Medical Ltd, Caesarea, Israel) to evaluate flow-mediated increases in pulse amplitude. Endo-PAT results are expressed as the natural logarithm of the ratio of the pulse amplitude recorded 90–120 s after cuff release to the baseline amplitude divided by the hyperemic to baseline ratio in the contralateral control finger (lnPAT ratio). A higher lnPAT ratio reflects better endothelial function. Ultrasound images of the brachial artery (Powervision 6000, Toshiba Medical, Inc., Tustin, CA) were digitized online using customized hardware (Cardiovascular Engineering, Inc., Holliston, MA) and then analyzed using the Brachial Analyzer (Medical Imaging Applications, Iowa City, IA). The stiffness of the central aorta and conduit arteries was also assessed in the upper extremity by measuring carotid-femoral and carotid-radial pulse wave velocity, respectively, using an applanation tonometry device (Complior SP, Artech Medical, Pantin, France) which calculated pulse wave velocity (m/s) [17]. To assess treatment effects on the endothelium-independent vasodilation of the brachial artery, nitroglycerin (0.4 mg sublingual)-mediated vasodilation of the brachial artery was assessed as previously described [18, 19]. The nitroglycerin portion of the study was omitted if systolic blood pressure was <100 mmHg, if the subject had a history of migraine headaches, or if he/she reported a previous history of prior adverse reaction to nitroglycerin. Thus, the sample size was smaller for nitroglycerin mediated dilation. Subjects were asked to take their last dose of medication 24 h before each visit, and if applicable, to avoid smoking on the morning of the study.

### Biochemical biomarkers

Whole blood samples were separated by centrifugation (1,000 × g, 15 min), and plasma and serum were stored at −80 °C until use. Plasma concentrations of total cholesterol (TC), high-density lipoprotein-cholesterol (HDL-C), and triglycerides (TG) were determined with a clinical chemistry analyzer (Olympus AU400, Center Valley, PA) according to the manufacturer’s instructions. The intra- and inter-day coefficients of variation (CV) for TC were 1.6 and 2.8 %, for HDL-C was 3.0 and 7.0 %, and for TG was 2.0 and 3.4 %, respectively. Inflammatory status was evaluated by plasma concentrations of interleukin (IL)-6 and tumor necrosis factor (TNF) α, which were assessed with a high sensitivity Quantikine quantitative sandwich enzyme immunoassay (R&D Systems, Minneapolis, MN) with intra- and inter-day CV of 6.9 and 6.5 % and of 5.3 and 10.8 %, respectively. High sensitivity C-reactive protein (CRP) was determined by a chemiluminescent immunometric assay (IMMULITE 1000, Siemens Healthcare Diagnostics, Los Angeles, CA) with intra- and inter-day CV of 4.2 and 4.8 %, respectively. Plasma and urinary nitric oxide (NO) were determined using a commercial assay kit [Nitrate/Nitrite Colorimetric Assay Kit Lactate Dehydrogenase (LDH) Method, Assay Designs, Ann Arbor, MI]. The intra- and inter-day CV for plasma was 7 and 7.6 % and for urine 5.3 and 4.7 %, respectively. Vascular cell adhesion molecule-1 (VCAM-1) and E-selectin concentrations in plasma were determined by an enzyme-linked immunosorbent assay (ELISA) kit (R&D Systems and eBioscience, San Diego, CA, respectively). The intra- and inter-day CV was 4.4 and 2.3 % for VCAM-1 and 8.8 and 10.6 % for E-selectin, respectively. Circulating oxidized LDL in plasma was determined by an ELISA kit (ALPCO, Salem, NH) with intra- and inter-day CV of 7.2 and 10.6 %, respectively. A reverse-phase gradient high performance liquid chromatography (HPLC) method was used for the simultaneous determination of plasma α- and γ-tocopherol [20]. The intra- and inter-day CV were 3.8 and 6.4 % for α-tocopherol and 3.1 and 6.1 % for γ-tocopherol, respectively. ApoA-I, ApoB, direct LDL-C, and small dense LDL-C were measured employing an Olympus AU400 auomated analyzer using kits (Kamiya Biomedical Company, Seattle, WA; Beckman Coulter, Inc., Brea, CA; and Randox Laboratories, Ltd. Crumin, UK, respectively).

### Dietary assessment

Dietary intake was estimated using a self-administered semi-quantitative food frequency questionnaire (FFQ) which was validated by Willett and Hu [21] before the run-in and at the end of each treatment period. The FFQ included 161 food items with a standard portion included as part of the question and food-specific frequencies. In addition, the questionnaire included questions on dietary supplement intake, types of fats and cooking methods and an open-ended question to include regularly consumed foods not captured in the food list. The FFQ was analyzed by the Harvard School of Public Health and daily nutrient intakes were provided. Almonds consumed during the intervention phase were not recorded in the questionnaire.

### Statistical analysis

Results are expressed as mean ± standard deviation (SD). A repeated measures analysis was performed to analyze significance between treatments using a general linear model with PROC GLM with treatment (ALM vs. CON), sequence (ALM-CON vs. CON-ALM), period (1 vs. 2), and subjects as variables, followed by using LSMEANS to evaluate the significance in differences between the ALM and CON diets. The difference in nutrient intakes between before the run-in and at the end of each intervention phase was assessed using the Student’s *t*-test. Significance was considered at *P* ≤0.05. All statistical analyses were performed using SAS version 9.3 (SAS Institute Inc., Cary, NC).

The sample size of this crossover study was obtained on the basis of prior data from our laboratory with a power of 88 % (α = 0.05) to detect a 1 % change in the pre-specified primary endpoint of brachial artery FMD (e.g., from 6.0 to 7.0 %) [15].

## Results

### Subjects

Forty five subjects completed the trial (demographic information presented in Table 1). The mean systolic blood pressure was 3.2 mmHg lower than the definition of stage 1 hypertension and the mean diastolic blood pressure was within the normal range. Mean TC, LDL-C, HDL-C, and TG values were all in the normal range. All subjects were treated with multiple medications during the trial. The average number of medications used before and after the ALM diet and before and after the CON diet were 7.9 ± 3.3 (range: 3–20), 8.1 ± 3.8 (3–24), 8.2 ± 3.7 (3–24), and 8.1 ± 3.8 (3–26), respectively. The number and type of medications were stable during the study and not significantly different between the treatment periods. The classes of drugs used by the subjects (as percent of the 45 subjects) were: aspirin (100), statins (96), vasodilators (87), beta-blockers (84), hypogycemics (82), platelet inhibitors (44), angiotensin converting enzyme inhibitors (38), diuretics (33), proton pump inhibitors (24), and calcium channel blockers (22). The dose of the medications was not monitored.

**Table 1:** Characteristics of study participants at screening*

Attribute	Value
Sex, F/M	27/18
Age, year	61.8 ± 8.6
Body weight, kg	86.6 ± 14.6
Body mass index, kg/m^2^	30.2 ± 5.1
Waist, cm (in)	102.8 ± 13.3 (40.5 ± 5.2)
Total cholesterol, mg/dL	153.4 ± 37.0
HDL-cholesterol, mg/dL	43.9 ± 11.1
LDL-cholesterol, mg/dL	82.6 ± 28.2
Total triglycerides, mg/dL	132.7 ± 82.3
Systolic Pressure, mmHg	136.8 ± 21.4
Diastolic Pressure, mmHg	74.5 ± 10.7

*Only completers are included

### Nutrient intake and status

The incorporation of almonds into the CON diet increased the intake of vitamin E by 103 % compared to CON diet. The ALM diet also increased plasma α-tocopherol concentration by 5.8 % and decreased γ-tocopherol by 15.4 % compared to the baseline value of the almond phase, whereas CON diet did not have a significant impact on either nutrient (Table 2). Compared to the CON diet, the ALM diet increased the post-intervention value of plasma α-tocopherol by 10.2 % (*P* = 0.0065) and decreased γ-tocopherol by 16.5 % (*P* = 0.047).

**Table 2:** The change in nutrient consumption before the run-in and at the end of the 2 interventions^a^

	Before run-in	Almond diet	Control diet
Calories, Kcal	1732.9 ± 859.1	2049.8 ± 733.0^bd^	1579.6 ± 784.8^c^
Proteins, g	70.5 ± 32.3	86.6 ± 33.1^bd^	67.1 ± 28.4^c^
Animal protein, g	44.3 ± 21.7	45.4 ± 24.8	43.9 ± 19.4
Protein, % Kcal	16.8 ± 3.6	16.9 ± 2.6	17.6 ± 3.3
Carbohydrates, g	232.0 ± 28.7	215.4 ± 101.1	215.3 ± 122.8
Carbohydrate, % Kcal	53.4 ± 7.3	40.7 ± 7.8^bd^	53.2 ± 8.3^c^
Total Fats, g	59.9 ± 37.3	98.1 ± 30.8^b^	50.6 ± 27.9^c^
Fat % Kcal	30.5 ± 6.0	44.4 ± 7.2^bd^	29.1 ± 6.8^c^
SFA, g	19.7 ± 13.2	20.3 ± 10.1^b^	16.4 ± 9.4^cd^
SFA % Kcal	9.9 ± 2.8	8.8 ± 2.2^d^	9.4 ± 2.8
MUFA, g	21.6 ± 13.3	48.1 ± 12.0^bd^	18.3 ± 10.3^cd^
MUFA % Kcal	11.2 ± 2.5	22.4 ± 4.9^bd^	10.6 ± 3.0^c^
PUFA, g	12.9 ± 8.3	22.5 ± 7.0^bd^	11.0 ± 6.7^c^
PUFA % Kcal	6.6 ± 1.7	10.2 ± 1.6^bd^	6.3 ± 1.5^c^
Cholesterols, mg	201.8 ± 148.6	197.8 ± 133.3	188.2 ± 94.3
Fiber, g	19.7 ± 11.2	28.7 ± 10.0^bd^	18.7 ± 12.5^c^
Glucose, g	22.3 ± 15.3	18.5 ± 11.8	19.6 ± 13.9
Sucrose, g	42.6 ± 29.0	37.1 ± 24.4	39.1 ± 31.2
Fructose, g	23.6 ± 17.4	19.9 ± 13.5	21.5 ± 16.8
Alcohol, g	2.3 ± 3.7	3.1 ± 4.3	3.3 ± 4.9
Caffeine, mg	152.7 152.5	128.2 ± 117.0	133.7 ± 139.8
Vitamins			
Vitamin A, IU	10759.2 ± 6077.7	10612.5 ± 7059.1	10880.6 ± 7530.9
Vitamin C, mg	163.6 ± 183.5	117.7 ± 105.1	129.9 ± 108.3
Vitamin D, IU	405.9 ± 339.6	381.2 ± 372.2	397.3 ± 372.9
a-Tocopherol, mg	7.0 ± 3.7	34.5 ± 9.8^bd^	17.0 ± 29.4^cd^
Vitamin K, μg	143.1 ± 102.7	135.9 ± 85.5	150.2 ± 125.0
Vitamin B1, mg	3.3 ± 7.7	3.2 ± 7.7	3.1 ± 7.6
Vitamin B2, mg	4.0 ± 7.8	4.6 ± 7.8^b^	3.7 ± 7.7^c^
Niacin, mg	37.1 ± 40.2	37.1 ± 38.9	34.9 ± 38.9
Vitamin B6, mg	4.6 ± 8.6	5.7 ± 11.4	6.7 ± 17.1
Folate, μg	943.9 ± 568.3	567.4 ± 346.7^d^	580.1 ± 378.3^d^
Vitamin B12, μg	46.3 ± 127.8	57.8 ± 145.2	67.1 ± 161.2
Choline, mg	288.5 ± 140.4	325.7 ± 142.6^b^	274.2 ± 116.6^c^
Pantothenic acid, mg	10.6 ± 10.3	9.6 ± 10.3	9.7 ± 10.2
Retinol, IU	2767.5 ± 1785.9	2388.0 ± 2813.2	2412.3 ± 1965.0
Phytochemicals			
Flavonoids, mg	252.2 ± 317.0	205.4 ± 252.7	204.7 ± 243.0
Proanthocyanins, mg	127.5 ± 90.9	275.2 ± 87.5^bd^	117.3 ± 114.8^c^
Carotenoids, mg	7991.7 ± 5219.5	8222.5 ± 5679.8	8459.3 ± 6667.4
Minerals			
Calcium, mg	982.1 ± 651.3	1078.5 ± 594.5^b^	865.5 ± 490.0^c^
Copper, mg	2.0 ± 1.3	2.8 ± 1.8^bd^	1.9 ± 1.5^c^
Iodine, μg	37.1 ± 63.5	37.1 ± 63.5	40.5 ± 72.8
Iron, mg	17.0 ± 11.3	19.0 ± 11.9	19.0 ± 16.0
Potassium, mg	2861.9 ± 1334.9	3261.2 ± 1329.2^b^	2697.2 ± 1312.5^c^
Magnesium, mg	345.2 ± 190.5	544.5 ± 167.9^bd^	319.9 ± 174.8^c^
Manganese, mg	4.1 ± 2.4	5.6 ± 2.4^bd^	4.0 ± 2.5^c^
Phosphate, mg	1234.0 ± 571.2	1537.5 ± 553.7^bd^	1144.2 ± 522.7^c^
Selenium, μg	5.0 ± 8.5	12.2 ± 22.4	10.3 ± 25.4
Zinc, mg	14.4 ± 8.9	16.9 ± 9.6^bd^	14.5 ± 11.2^c^
Amino acids			
Alanine, g	3.3 ± 1.6	4.2 ± 1.6^bd^	3.2 ± 1.3^c^
Arginine, g	3.9 ± 1.9	6.0 ± 1.8^bd^	3.7 ± 1.5b
Aspartic acid, g	6.5 ± 3.0	8.6 ± 3.0^bd^	6.1 ± 2.6^c^
Cysteine, g	1.1 ± 0.5	1.2 ± 0.5^b^	1.0 ± 0.5^c^
Glutamic acid, g	13.7 ± 6.3	18.3 ± 6.3^bd^	12.9 ± 5.7^c^
Glycine, g	3.0 ± 1.4	4.2 ± 1.4^bd^	2.8 ± 1.2^c^
Histidine, g	1.9 ± 0.9	2.4 ± 0.9^bc^	1.8 ± 0.8^c^
Isoleucine, g	3.2 ± 1.5	3.7 ± 1.5^b^	3.0 ± 1.3^c^
Leucine, g	5.6 ± 2.6	6.7 ± 2.7^b^	5.3 ± 2.2^c^
Lysine, g	4.7 ± 2.2	5.2 ± 2.4	4.6 ± 1.9
Methionine, g	1.6 ± 0.7	1.7 ± 0.8^b^	1.5 ± 0.6^c^
Phenylalanine, g	3.2 ± 1.4	4.0 ± 1.5^bd^	3.0 ± 1.3^c^
Proline, g	4.6 ± 2.2	5.0 ± 2.2^b^	4.2 ± 2.0^c^
Serine, g	3.3 ± 1.5	3.9 ± 1.5^b^	3.1 ± 1.3^c^
Threonine, g	2.6 ± 1.2	3.1 ± 1.2^b^	2.5 ± 1.0
Tryptophan, g	0.8 ± 0.3	0.9 ± 0.3^b^	0.7 ± 0.3^c^
Tyrosine, g	2.4 ± 1.1	2.7 ± 1.2^b^	2.3 ± 1.0^c^
Valine, g	3.7 ± 1.7	4.3 ± 1.7^b^	3.5 ± 1.5^c^

^a^Abbreviation: *CHO* carbohydrates, *SFA* saturated fatty acids, *MUFA* monounsaturated fatty acids, *PUFA* polyunsaturated fatty acids

^bc^Means with different letters between almond and control diets differ, tested by LSMEANS

^d^Means differ from those of before the run-in, tested by Student’s *t*-test

The addition of almonds to the CON diet increased energy intake by 30 %, mainly due to a 163 % increase in the intake of monounsaturated fatty acids (MUFA). The amount of carbohydrate intake did not differ between CON and ALM diets, but the percentage contribution of carbohydrates to total energy intake when consuming the ALM diet was 23 % lower than when consuming CON diet. The amount of protein consumption during the ALM phase was 29 % greater than that of the CON phase but the contribution of protein to total energy intake was not different. Fiber intake was increased by 53 % by ALM diet as compared to CON diet. The ALM diet led to 24, 19, and 135 % greater intake of riboflavin, choline, and proanthocyanins, respectively, than the CON diet. The ALM diet was also associated with a greater intake of calcium, copper, potassium, magnesium, manganese, phosphate, and zinc by 25, 47, 21, 70, 40, 34, and 17 % compared to CON diet, respectively. Almonds are rich in arginine and daily consumption of 85 g of the nuts increased the intake of this amino acid by 62 %. The intake of other amino acids (except lysine) was also increased by the ALM diet, ranging from 17 % in tyrosine to 240 % in methionine.

### Vascular reactivity

The main study objective was to determine whether 6 weeks of almond consumption would impact vascular reactivity. As shown in Table 3, we did not observe a significant effect of almonds incorporated to the NCEP step 1 diet or the NCEP step 1 diet alone on FMD, the principal pre-specified primary endpoint, or on blood pressure, nitroglycerin-mediated dilation, hyperemic blood flow, change in forearm blood flow, arterial stiffness, PWV, and lnPAT ratio.

**Table 3:** The effect of almonds on measures of vascular activity^a^

Biomarker	Almond diet		Control diet	
	Pre	Post	Pre	Post
SBP, mmHg	135 ± 21	135 ± 20	136 ± 26	135 ± 23
DBP, mmHg	73.3 ± 10.3	73.8 ± 9.5	74.1 ± 10.7	73.7 ± 10.6
FMD, %	7.7 ± 3.3	8.3 ± 3.8	7.8 ± 3.5	7.5 ± 3.7
NMD, %^b^	12.0 ± 5.4	11.3 ± 5.3	11.2 ± 5.4	10.4 ± 5.2
Carotid-femoral PWV, m/s	8.6 ± 1.6	8.4 ± 1.8	8.4 ± 1.7	8.4 ± 1.3
Carotid-radial PWV, m/s	8.7 ± 1.6	8.5 ± 1.6	8.7 ± 1.8	8.2 ± 1.4
Hyperemic blood flow, mL/min	984 ± 304	968 ± 334	1095 ± 477	964 ± 339
Post velocity, cm/s	114 ± 28	109 ± 27	131 ± 99	107 ± 28
Change in FBF (%)	657 ± 301	582 ± 221	592 ± 210	603 ± 227
LnPAT	0.61 ± 0.39	0.43 ± 0.47	0.47 ± 0.46	0.50 ± 0.40

^a^*Abbrevations*: *DBP* diastolic blood pressure, *FBF* forearm blood flow, *FMD* flow mediated dilation, *LnPAT* natural log of the pulse amplitude tonometry measured in the finger, *PWV* pulse wave velocity, *SBP* systolic blood pressure

^b^Nitroglycerin-mediated dilation (NMD) of the brachial artery, *n* = 22 participants because some subjects did not participate in this portion of the study if their SBP was <100 mmHg, if they had a history of migraine headaches or if they reported a previous history of prior adverse reaction to nitroglycerin

### Biochemical biomarkers

The lipid profiles of the subjects, including TC, HDL-C, LDL-C, TG, and small density LDL-C, was unchanged during the study (Table 4). VLDL-C values were calculated from TG concentration, so changes in VLDL-C were identical to those in observed in TG. The building block of LDL and HDL, apoprotein-B100 and -AI, respectively, was not altered by either diet. Biomarkers of inflammation, including plasma CRP, IL-6, and TNFα, were also not affected by either diet. Endothelial function was evaluated plasma E-selectin, VCAM-1, and NO. E-selectin and NO levels were not affected by either diet. As compared to the CON diet, the ALM diet tended to decrease sVCAM-1 by 6.1 % (ALM vs. CON: 787 vs. 835 ng/mL, *P* = 0.064). Urinary NO at the post-ALM diet period was 17.5 % greater than that of the post-CON diet (0.56 vs. 0.47 μmol/mg creatinine, *P* = 0.09).

**Table 4:** Effect of almonds on biochemical biomarkers^a^

Biomarker	Almond diet		Control diet	
	Pre	Post	Pre	Post
Plasma Lipid Profile				
Total cholesterol, mg/dL	146.2 ± 37.8	146.9 ± 32.4	149.8 ± 43.2	148.6 ± 38.3
HDL-C, mg/dL	41.0 ± 11.2	41.7 ± 11.2	41.7 ± 10.8	43.3 ± 15.6
LDL-C, mg/dL	78.7 ± 28.6	80.3 ± 26.2	82.67 ± 36.3	77.9 ± 26.3
Total triglycerides, mg/dL	123.6 ± 90.9	124.3 ± 60.7	129.2 ± 75.5	131.4 ± 60.2
Small dense LDL-C, mg/dL	30.9 ± 14.1	32.8 ± 14.1	33.7 ± 19.9	29.6 ± 12.1
Apo-B100, mg/dL	69.0 ± 20.8	71.9 ± 19.0	72.3 ± 25.0	67.1 ± 19.8
Apo-AI, mg/dL	118.0 ± 22.0	118.4 ± 22.1	118.8 ± 21.0	121.0 ± 28.1
Plasma Inflammatory cytokines				
C-reactive Protein, mg/L	4.0 ± 6.4	3.3 ± 4.2	4.5 ± 5.7	3.9 ± 5.1
TNFα, pg/mL	1.8 ± 1.8	1.8 ± 1.6	1.6 ± 1.4	1.7 ± 1.5
IL-6, pg/mL	3.5 ± 2.2	3.6 ± 2.5	3.8 ± 2.9	4.1 ± 2.9
Endothelial function				
Plasma E-selectin, ng/mL	28.7 ± 19.6	28.7 ± 18.6	29.4 ± 20.7	27.5 ± 17.7
Plasma VCAM-1, ng/mL	854 ± 377	787 ± 363^b^	826 ± 380	835 ± 341
Plasma nitric oxide, μmol/L	38.2 ± 12.6	41.8 ± 15.8	42.1 ± 17.4	39.3 ± 13.1
Urinary nitric oxide, μmol/mg creatinine	0.62 ± 0.59	0.56 ± 0.36^c^	0.63 ± 0.33	0.47 ± 0.29
Oxidative stress				
Circulating oxidized LDL, mU/mL	741 ± 704	727 ± 637	729 ± 604	778 ± 746
α-Tocopherol, μg/dL	1080 ± 453	1143 ± 388**	1120 ± 362	1037 ± 347
γ-Tocopherol, μg/dL	156 ± 82	132 ± 69*	175 ± 104	158 ± 84

^a^Abbreviation: *VCAM-1* vascular cell adhesion molecule-1

^b^*P* = 0.0641, ^c^*P* = 0.09, *P <0.05, **P <0.01, statistical significance between post values tested by LSMEANS

## Discussion

This randomized, crossover clinical trial was designed to examine the impact of almonds incorporated to a NCEP step 1 diet on vascular reactivity and other CVD risk factors in patients with CAD, using the NCEP step 1 diet without nuts as the control phase. Neither the ALM nor CON diet affected measures of endothelial vasodilator function, including FMD, lnPAT ratio, and PWV, a measure of arterial stiffness. In addition, no significant effect of ALM compared to CON on the plasma lipid profile or selected biomarkers of inflammation, endothelial function, and oxidative stress was observed.

Endothelial dysfunction, a critical early event in the pathogenesis of atherosclerosis characterized by the reduced bioavailability of NO (a vasodilator) and the increased expression of cellular adhesion molecules, is related to perfusion abnormalities and subsequent ischemic events [22]. However, we found no beneficial effect of chronic almond consumption on vascular reactivity reflected by FMD, NMD, lnPAT ratio, and PWV, even though the ALM diet significantly increased intake of several nutrients associated with improved endothelial function in other studies. Our results are consistent with the study by López-Uriarte et al. [23] in which a 12-weeks intervention study with mixed nuts (15 g walnuts, 7.5 g almonds, and 7.5 g hazelnuts) did not show an effect on endothelial function assessed by PAT in patients with Metabolic Syndrome. In contrast, Choudhury et al. [14] reported that 50 g almonds/day for 4 weeks improved FMD asymptomatic men as compared to no almonds (3.6 vs. 2.9 %). Similarly, a 4-weeks intervention where hazelnuts replaced ~20 % daily energy intake significantly improved FMD (56.6 %) from 15.2 to 21.8 % as compared to control in 21 adults with hypercholesterolemia [24]. Further, Katz et al. [25] found the intake of 56 g/day walnuts for 8 weeks significantly increased FMD from 8.8 to 10.2 % in overweight patients with Metabolic Syndrome, accompanied by a reduction in systolic blood pressure. Ros et al. [26] also reported that walnuts incorporated into a cholesterol-lowering Mediterranean diet to replace 32 % MUFA energy improved endothelium-dependent FMD from 4.1 to 5.1 % in subjects with hypercholesterolemia. Differences in the study design, subject characteristics (including health status, lifestyle, and drug therapy), and nut nutrient composition may account for the discordant results obtained here. For example, all of our patients were receiving multiple risk-reduction therapies and had well-controlled lipid profiles at baseline. The discrepant results might also reflect differences in the nutrient content of the various tree nuts; e.g., walnuts contain more polyunsaturated fats, including α-linoleic acid, than MUFA compared to other tree nuts. However, the exact mechanism(s) for the discrepancy between our studies and others remain to be explored in the future studies.

The effect of chronic consumption of almonds and other tree nuts on blood biomarkers of CVD have been demonstrated, although the results are mixed with regard to individual biomarkers. Previously, we observed that almonds replacing 20 % daily caloric intake in a NCEP step 2 diet improved lipid profile in Chinese patients with type 2 diabetes [27]. The benefits of almonds on the lipid profile of healthy adults or those who have hypercholesterolemia, dyslipidemia, and/or prediabetes have been reported in several studies [28–33]. More recently, Nishi et al. [34] found that almond consumption at 37 or 73 g/day for 4 weeks increased oleic acid and MUFA content in serum TG and non-esterified fatty acid fractions. Jenkins et al. [16] extrapolated from their dose–response study to suggest that each 7-g portion of almonds would induce a 1 % reduction in LDL-C. In contrast, we did not find that the addition of almonds to the NCEP step 1 diet affected TC, TG, HDL-C, LDL-C or small density LDL-C. However, as the lipid profile at study baseline in our subjects were being maintained within normal ranges by statins and/or the NCEP Step 1 diet, it seems unlikely that any single food would enable a further significant improvement.

Inflammation plays a critical role in the risk for and progression of CVD such that inflammatory biomarkers like CRP and IL-6 are generally recognized as independent predictors of atherosclerosis [35–37]. Cell adhesion molecules, including VCAM-1, E-selectin, and intercellular adhesion molecule-1 (ICAM-1), also contribute to the pathogenesis of this condition [38]. Jiang et al. [39] noted in the Multi-Ethnic Study of Atherosclerosis that inflammatory biomarkers were inversely associated with an increased frequency of nut and seed consumption. Consistent with this observational data, we previously found that almonds decreased IL-6 and CRP, and TNF-α in Chinese patients with type 2 diabetes but did not affect ICAM-1 or VCAM-1 [40]. Rajaram et al. [41] also reported that almond intake diminished CRP in free-living healthy adults although no dose–response relationship was noted. In contrast, Damasceno et al. [28] reported that neither almonds nor walnuts affected CRP, ICAM-1 or VCAM-1 in asymptomatic adults with moderate hypercholesterolemia. In our study of well-controlled CAD patients, neither the ALM nor the CON diet had an impact on IL-6, CRP, TNF-α or E-selectin. However, there was a trend toward reduction in VCAM-1 after the ALM phase. It is noteworthy that the concentration of CRP, IL-6 and TNF observed in this study appeared to be slightly larger than the values found in Chinese patients with type 2 diabetes [27] or coronary heart disease [42] and in the prospective Multi-Ethnic Study of Atherosclerosis [39]. Thus, it is plausible that the larger starting values in inflammatory biomarkers may account for the observed null results. Also, the null effect may be attributed to the direct or indirect anti-inflammatory effects of multiple medications taken by the patients. Similar to the slight improvement in VCAM-1, urinary excretion of NO tended to increase with the ALM diet. These trends suggest a possible modest benefit of almond intake on endothelial function in CAD patients, so further research on this potential benefit is warranted.

Almonds are a good source for a number of nutrients, with 85 g providing >10 % of the daily value of many vitamins (vitamin E, riboflavin, niacin, thiamin, and folate), minerals (calcium, copper, iron, magnesium, manganese, phosphorus, potassium, zinc), and fiber as well as arginine and oleic acid [43]. In addition, 85 g almonds contain 111 mg β-sistosterol, 9.3 mg flavonoids, and 156.5 mg proanthocyanins [44]. Relative to the CON diet, the ALM diet had a more positive impact on nutritional quality by increasing intake of calcium, magnesium, choline, and fiber above recommended values. The intake of these nutrients were below either the EAR or RDA when the subjects were consuming CON diet. These results are consistent with Jaceldo-Siegl et al. [45] who found the addition of 52 g/day almonds to the habitual diets of healthy men and women for 6 months significantly increased their intakes of MUFA, PUFA, fiber, vegetable protein, vitamin E, copper, and magnesium and decreased the amount of *trans* fatty acids, animal protein, sodium, cholesterol, and sugars. Consumption of dietary polyphenols is associated inversely with the risk of CVD and cancer [46–48]. As almonds contain an array of polyphenols, particularly proanthocyanins, they can be part of recommended guidelines for healthy diets rich in plant foods.

The study participants were patients with angiographically proven CAD and were taking on average 8 medications daily. Their polypharmacy regimen remained stable and effective during the 6-weeks almond intervention, suggesting intakes at 85 g/day are absent any indication of untoward drug-food interactions. Importantly, the change in the profile of nutrient intake during the ALM phase matched well general dietary recommendations to reduce the risk of CVD. It is also noteworthy that even though the ALM diet increased the percent of total calorie intake from fat from 29.1 to 44.4 %, there was no adverse effect on the lipid profile. These results are consistent with the favorable effect of almonds on lipid profiles reported by others [49].

Our study had several limitations. The study duration of 6 weeks for each arm is a relatively short period to affect some biomarkers of CVD risk and progression, so a longer intervention or larger sample size may have resulted in a statistically significant reduction in plasma VCAM-1 and increase in urinary NO. Conversely, while we found no untoward impact of the ALM diet on concurrent drug therapy, we cannot exclude the development of such interactions over a longer period. It is possible the study design, including a 6-weeks run-in period on the NCEP Step 1 diet that was then continued throughout both study arms and the 4-weeks washout, may have masked an impact of the almond intervention. Similarly, efficacy of the polypharmacy regimens of the subjects may have largely achieved the maximal benefit possible in these patients on the outcome parameters of vascular reactivity, inflammation, and oxidative stress. Finally, the study might be underpowered for both the primary and secondary outcome measures since the power calculation was performed based on the improvement in FMD.

## Conclusions

This randomized, controlled, cross-over trial revealed no significant impact of the incorporation of 85 g/day almonds to a NCEP Step 1 diet on FMD and other measures of vascular reactivity. Compliance to the almond intervention was good, as confirmed by an elevation of α-tocopherol status, and was associated with an improvement of dietary quality reflected by increased intakes of fiber, amino acids (especially arginine), the minerals calcium and magnesium, choline, and proanthocyanins. Positive trends were observed in a reduction of circulating VCAM-1 and an increase of urinary NO, though these changes did not achieve statistical significance. The addition of almonds to the control diet improved dietary quality beyond that already achieved by the NCEP step 1 diet, particularly by increasing the intake of fiber, calcium, magnesium, choline, proanthocyanins, and arginine. The generous consumption of almonds was safe in CAD patients, absent any indication of adverse interactions with their polypharmacy regimens. Overall, our results provide further support for patients with CVD that almonds can be part of a healthy diet.

## Abbreviations

ALM: Almond diet
BMI: Body mass index
CON: NCEP step I diet
CRP: C-reactive protein
CVD: Cardiovascular disease
CV: Coefficients of variation
CAD: Coronary artery disease
FMD: Flow-mediated dilatation
FFQ: Food frequency questionnaire
HDL-C: High-density lipoprotein-cholesterol
HPLC: High performance liquid chromatography
ICAM-1: Intercellular adhesion molecule-1
IL-6: Interleukin-6
LDL-C: Low-density lipoprotein-cholesterol
MUFA: Monounsaturated fatty acids
NCEP: National Cholesterol Education Program
NO: Nitric oxide
PAT: Peripheral arterial tonometry
PWV: Pulse wave velocity
TC: Total cholesterol
TG: Triglycerides
TNF-α: Tumor necrosis factor-α
VCAM-1: Vascular cell adhesion molecule-1
VLDL-C: Very low-density lipoprotein-cholesterol
